# Are women in Singapore gaining weight appropriately during pregnancy: a prospective cohort study

**DOI:** 10.1186/s12884-019-2443-z

**Published:** 2019-08-13

**Authors:** Song He, John Carson Allen, Nurul Syaza Razali, Nyo Mie Win, Jun Jim Zhang, Mor Jack Ng, George Seow Heong Yeo, Bernard Su Min Chern, Kok Hian Tan

**Affiliations:** 1 Division of Obstetrics and Gynaecology, KK Women’s and Children’s Hospital, Singapore, Singapore; 2 Office of Clinical Sciences, Duke-NUS Medical School, Singapore, Singapore; 3 Division of Obstetrics and Gynaecology, OBGYN Academic Clinical Program (ACP), KK Women’s and Children’s Hospital, Singapore, Singapore; 4 Department of Maternal Fetal Medicine, KK Women’s and Children’s Hospital, 100 Bukit Timah Road, Singapore, 229899, Singapore

**Keywords:** Gestational weight gain, Overweight, Obesity, Pregnancy

## Abstract

**Background:**

We aimed to study gestational weight gain (GWG) in a Singaporean population and compare it with Institute of Medicine (IOM) 2009 GWG guidelines.

**Methods:**

Nine hundred twenty-six women with low-risk singleton pregnancy were enrolled in a prospective cohort study from 2010 to 2014 in a Singapore tertiary maternity hospital. Seven hundred twenty-four patients had maternal weight information till term pregnancy and were included in analysis. Participants were categorized according to their first antenatal visit body mass index (BMI) as underweight, normal weight, overweight and obese. Total GWG for each BMI group was calculated. Multivariate logistic regression was performed to determine the predictors of total GWG above and below IOM guidelines.

**Results:**

Obese women had a mean total GWG (9.1 kg) that exceeded the upper limit IOM guidelines (9 kg). In multivariate analysis of predictors of total GWG above IOM guidelines, being overweight (adjusted OR: 3.91 [95% CI, 2.60–5.88]; *p* < .0001) and obese (adjusted OR: 4.78 [95% CI, 2.80, 8.15]; p < .0001) significantly increased the risks of gaining weight above IOM guidelines during pregnancy, compared to being normal weight.

**Conclusions:**

Overweight and obesity are independent significant risk factors for gaining excessive gestational weight. Appropriate weight management for overweight and obese Singaporean women prior to and during pregnancy is important.

**Electronic supplementary material:**

The online version of this article (10.1186/s12884-019-2443-z) contains supplementary material, which is available to authorized users.

## Background

Inadequate or excessive gestational weight gain (GWG) is associated with adverse pregnancy and neonatal outcomes [1]. For example, studies have shown associations between excessive GWG and increased birth weight and postpartum weight retention [2]. On the other hand, inadequate GWG is associated with higher risks of small for gestational age and preterm birth [1].

In 2009, the Institute of Medicine (IOM) published revised guidelines on GWG based on short-term and long-term consequences of GWG on maternal, fetal and child health outcomes. The IOM 2009 guidelines are based on maternal pre-pregnancy body mass index (BMI) as per the World Health Organization (WHO) classification and are independent of age, race, parity, smoking and ethnic background [3]. Many population-based studies have since compared GWG with IOM 2009 guidelines and most showed low adherent rates to the IOM recommendation [4].

Thus far, most studies on the characteristics of GWG were done in Western countries [5–8]. The majority of studies on GWG in Asian populations were conducted in China and Japan [9–14]. As there is limited data on the characteristics of pregnancy weight gain in the Southeast Asia, we would like to explore this in the Singaporean population. The objective of our study is to examine in our cohort the predictors of GWG above and below IOM 2009 guidelines, to analyze total GWG and rate of GWG in the second and third trimesters and compare it with IOM guidelines as well as other international and Asian-centered population studies.

## Methods

### Study design, setting and participants

The current study is nested in a prospective cohort study, known as the Neonatal and Obstetric Risk Assessment (NORA) study, which was conducted from September 2010 to August 2014 at the KK Women’s and Children’s Hospital, Singapore.

The study design of NORA has previously been described [15]. In summary, women attending their first antenatal visits in KK Women’s and Children’s Hospital, Singapore, were invited to participate in the NORA study. Those with viable singleton pregnancies confirmed by ultrasonography and at less than 14 weeks of amenorrhea at the first antenatal visit were eligible. The exclusion criteria were multiple pregnancies, chronic medical conditions that are associated with adverse pregnancy outcomes (such as systemic lupus erythematosus and renal diseases), pregnancies complicated by aneuploidy or fetal anomalies, and pregnancies ending in termination, miscarriage or fetal death before gestation age (GA) of 24 weeks. All participants gave written informed consent before the study began. One thousand thirteen patients participated in NORA and 926 completed the study. Of the 926 patients, 724 patients had maternal body weight information till term pregnancy (≥ 37 weeks) and were included in statistical analysis. Approval of the NORA study was obtained from the SingHealth Centralised Institutional Review Board Ethics Committee, Singapore (CIRB Ref No. 2010/214/D).

### GWG measurement

All women had body weight and height measured by clinic assistants during their first antenatal visits and the numbers were documented in the women’s medical records. First visit BMI was calculated as the first visit weight (in kg)/height squared (in m^2^). First visit BMI was used as an estimate of pre-pregnancy BMI with the assumption that there is negligible weight gain in the first trimester [16]. First visit BMI is categorized according to WHO BMI classification as follows: underweight (BMI < 18.5 kg/m^2^), normal weight (18.5 kg/m^2^ ≤ BMI < 25 kg/m^2^), overweight (25 kg/m^2^ ≤ BMI < 30 kg/m^2^), and obese (BMI ≥ 30 kg/m^2^). A breakdown by class of obesity is also included in the analysis of total GWG compared to IOM guidelines: Class I obesity BMI (30 kg/m^2^ ≤ BMI < 35 kg/m^2^), Class II obesity (35 kg/m^2^ ≤ BMI < 40 kg/m^2^), and Class III obesity (BMI ≥ 40 kg/m^2^).

Alongside patients’ routine antenatal visits, NORA comprised four stages of scheduled visits at 11–14 weeks (Visit 1), 18–22 weeks (Visit 2), 28–32 weeks (Visit 3) and 34–39 weeks (Visit 4). Maternal body weight and the corresponding GA were collected at the four scheduled NORA visits and measured during participants’ routine antenatal visits.

Total GWG was calculated as the difference between the first antenatal visit weight and the last documented weight during pregnancy (≥ 37 weeks). Rate of GWG (in kg/week) was calculated between different NORA visits and is defined as follows: rate of GWG 1 is the rate of GWG between scheduled Visit 2 and Visit 1, rate of GWG 2 is the rate of GWG between scheduled Visit 3 and Visit 2, and rate of GWG 3 is the rate of GWG between scheduled Visit 4 and Visit 3. These were calculated as (difference in weight /difference in GA) between the corresponding visits.

### Comparison with IOM 2009 GWG guidelines

The mean value of total GWG for each BMI group (as well as each subgroup of obesity) was compared to IOM 2009 GWG guidelines. According to IOM guidelines, the optimal range of total GWG in kg is 12.5–18, 11.5–16, 7–11.5, and 5–9 for the underweight, normal weight, overweight and obese group, respectively [3]. Similarly, the rate of GWG is compared to IOM 2009 guidelines which suggest mean (range) in kg/week in the second and third trimesters to be 0.51 (0.44–0.58), 0.42 (0.35–0.50), 0.28 (0.23–0.33), and 0.22 (0.17–0.27) for underweight, normal weight, overweight and obese women, respectively [4].

### Statistical methods

Demographic data and characteristics of study participants were summarized as mean ± SD for continuous variables and as percentages for categorical variables. Univariate and multivariate logistic regressions were used to assess the association between maternal demographics and baseline characteristics (age, race, BMI, parity, marital status, maternal employment status at onset of pregnancy, maternal educational level and monthly household income), behavioral factors (smoking, drinking and self-reported exercise), pre-existing medical conditions (pre-existing hypertension, pre-existing diabetes mellitus and thyroid disease) and illnesses during pregnancy (gestational diabetes, hypertensive disorders of pregnancy which includes pregnancy induced hypertension, pre-eclampsia and HELLP syndrome) with total GWG for the whole cohort and for each BMI group. SAS 9.3 Software (SAS, Inc., Cary, NC) was used for all statistical analyses. Statistical significance was set at *p* < 0.05.

## Results

### Population demographics and baseline characteristics

Table 1 summarizes the baseline characteristics of whole cohort. In our cohort, 8.1% were underweight, 57.2% normal weight, 23.9% overweight and 10.8% obese.

**Table 1:** Baseline characteristics of the whole cohort

Characteristics	Whole cohort (*N* = 724)	Characteristics	Whole cohort (N = 724)
Demographic factors		Maternal educational level, n (%)	
Maternal age (year), mean (SD)	30.6 (5.0)	Secondary and below	164 (22.7)
Maternal age range (year), n (%)		ITE ^a^	73 (10.1)
< 25	77 (10.6)	Junior college/Polytechnic	211 (29.2)
25–29	235 (32.5)	University and above	274 (37.9)
30–34	248 (34.3)	Total monthly household income ^b^, n (%)	
35–40	137 (18.9)	≤ 1300	24 (3.3)
> 40	27 (3.7)	1301–3500	227 (31.4)
Maternal body mass index (kg/m^2^), mean (SD)	23.9 (4.6)	3501–5500	218 (30.2)
Race, n (%)		5500–8500	163 (22.6)
Chinese	378 (52.2)	≥ 8501	90 (12.5)
Malay	120 (16.6)	Pre-pregnancy medical conditions	
Indian	141 (19.5)	Pre-existing hypertension, n (%)	9 (1.2)
Others	85 (11.7)	Pre-existing diabetes mellitus, n (%)	12 (1.7)
Parity, n (%)		Thyroid disease, n (%)	18 (2.5)
0	404 (55.8)	Behavioral factors	
1	225 (31.1)	Smoking, n (%)	
2 or more	95 (13.1)	Before current pregnancy	109 (15.1)
Maternal employment at onset of pregnancy, n (%)		During current pregnancy	17 (2.4)
Employed	581 (80.3)	Alcohol use, n (%)	
Unemployed	143 (19.7)	Before current pregnancy	219 (30.3)
Marital status, n (%)		During current pregnancy	9 (1.2)
Married	674 (93.1)	Exercise, n (%)	
Single	46 (6.4)	Before current pregnancy	341 (47.1)
Separated/Divorced	4 (0.5)	During current pregnancy	61 (8.4)

^a^ ITE stands for Institute of Technical Education. It is a public vocational education institution in Singapore that provides pre-employment training to secondary school leavers and continuing education and training to working adults.

^b^Total monthly household income is measured in Singapore dollars, and defined as the combined monthly income for married couple, or patient’s monthly income if she is single, separated or divorced.

Participants were categorized according to their first visit BMI. Baseline characteristics were analyzed for each BMI group and compared among the groups. These results were presented in Additional file 1: Table S1 “Baseline characteristics of each BMI groups”.

### Characteristics of GWG and comparison with IOM 2009 GWG guidelines

The mean (SD) of total GWG for the entire cohort was 11.8 (4.5) kg. The means of total GWG were within the IOM references for the underweight, normal weight and overweight women. In contrast, obese women had a mean (SD) total GWG of 9.1 (6.1) kg, which exceeded the upper limit of IOM recommendation (9 kg) (Table 2). Compared to IOM 2009 guidelines, 40.7% of women in our cohort had total GWG within recommendation, 33% gained less weight and 26.2% gained more weight than IOM recommendation. When analyzed by BMI subgroups, more underweight and normal weight women achieved total GWG within IOM guidelines, compared to overweight and obese women. In addition, higher proportions of obese and overweight women gained more weight than IOM references (Table 2).

**Table 2:** Total gestational weight gain with reference to IOM recommendations for different BMI groups

Pre-pregnancy BMI groups	IOM recommendation of total GWG (range in kg)	Total GWG in kg mean (SD) median (IQR)	Weight Gain <recommendation n (%)	Weight gain within recommendation n (%)	Weight gain >recommendation n (%)
Under Weight (*N* = 59)	12.5–18	12.7 (3.8) 13.00 (10.1, 14.6)	26 (44.1)	31 (52.5)	2 (3.4)
Normal Weight (*N* = 414)	11.5–16	12.5 (3.9) 12.6 (9.8, 15.0)	162 (39.1)	181 (43.7)	71 (17.2)
Over Weight (*N* = 173)	7–11.5	11.3 (4.6) 10.8 (8.1, 14.8)	28 (16.2)	66 (38.2)	79 (45.7)
Obese (*N* = 78)	5–9	9.1 (6.1) 8.9 (3.9, 12.7)	23 (29.5)	17 (21.8)	38 (48.7)
Class I Obesity (*n* = 61)		9.0 (5.8) 8.8 (4.0, 12.7)	17 (27.9)	15 (24.6)	29 (47.5)
Class II Obesity (*n* = 13)		7.1 (6.2) 6.2 (1.8, 12.7)	6 (46.2)	1 (7.7)	6 (46.2)
Class III Obesity (n = 4)		14.5 (8.7) 11.0 (9.7, 19.3)	0 (0)	1 (25.0)	3 (75.0)
Entire Cohort		11.8 (4.5) 11.8 (9.1, 14.7)	239 (33.0)	295 (40.7)	190 (26.3)

We further presented data for obesity subcategories separately to show the means of total GWG of each subcategory, and the percentages of women in each obesity subcategory that exceeded, achieved or gained weight below IOM target GWG. Although the number in each obesity subcategory is small, it appears that Class III obesity had a higher chance of gaining excessive weight than IOM guidelines, with a mean total GWG of 14.5 kg and 75% of the group had total GWG that exceeded IOM guidelines (Table 2).

The pattern of GWG for each BMI group is also characterized in terms of the average rate of GWG between different scheduled visits (in kg/week). Compared to IOM guidelines, overweight and obese women gained weight at higher rates than IOM recommendation between Visit 3 and Visit 2, i.e. between third and second trimesters, and between Visit 4 and Visit 3, i.e. within the third trimester. In contrast, underweight women had an average rate of weight gain within IOM recommendation between the third and second trimesters and in the third trimester, and normal weight women had an average rate of GWG within IOM guidelines in third trimester (Table 3).

**Table 3:** Rate of GWG (in kg/week) between scheduled visits with reference to IOM recommendations

Pre-pregnancy BMI groups	IOM recommendation of rate of GWG in 2nd and 3rd trimesters	GWG 1^a^	GWG 2^b^	GWG 3^c^
	Mean (range) in kg/week	Mean in kg/week (with ref. to IOM recommendation)		
Under Weight (N = 59)	0.51 (0.44–0.58)	0.38 (<R)	0.54 (WR)	0.50 (WR)
Normal Weight (N = 414)	0.42 (0.35–0.50)	0.33 (<R)	0.55 (>R)	0.47 (WR)
Over Weight (N = 173)	0.28 (0.23–0.33)	0.31 (WR)	0.47 (>R)	0.46 (>R)
Obese (N = 78)	0.22 (0.17–0.27)	0.19 (WR)	0.40 (>R)	0.46 (>R)
P-value comparing GWG among BMI groups		<.0001	<.0001	0.816

Visit 1 (11–14 Week); Visit 2 (18–24 Week); Visit 3 (28–32 Week); Visit 4 (34 Week Onwards)

Definitions: <R: weight Gain less than recommended range; WR: weight gain within recommended range;>R: weight gain more than recommended range

^a^GWG 1 = rate of gestational weight gain between scheduled Visit 2 and Visit 1

^b^GWG 2 = rate of gestational weight gain between scheduled Visit 3 and Visit 2

^c^GWG 3 = rate of gestational weight gain between scheduled Visit 4 and Visit 3

### Predictors of total GWG

Table 4 a summarizes the results of logistic regression on predictors of total GWG above IOM guidelines. Variables with a *p*-value < 0.20 in univariate analysis were entered in multivariate analysis. Maternal BMI category was significantly associated with total GWG above IOM recommendations, with underweight women less likely to gain weight above guidelines (adjusted OR: 0.25 [95% CI: 0.08–0.83]; *p* = 0.023), whereas overweight (adjusted OR: 3.91 [95% CI: 2.60–5.88]; *p* < .0001) and obese women (adjusted OR: 4.78 [95% CI, 2.80, 8.15]; p < .0001) had significantly increased risks of gaining weight above IOM guidelines compared to normal weight women. Maternal educational level also reached statistical significance; women with a university and above degree were less likely to gain weight above IOM guidelines. Additionally, women who smoked before pregnancy had a higher odds or gaining more weight than IOM references.

**Table 4:** Predictors of total gestational weight gain *above/below* IOM recommendations, whole cohort(N = 724)

4a) Logistic regression of predictors of total gestational weight gain *above* IOM recommendations				
Variable	Univariate OR ^a^		Multivariate Adjusted OR ^b^	
	Odds ratio (95% CI)	P-value	Odds ratio (95% CI)	P-value
Maternal body mass index category (kg/m^2^)		<.0001		<.0001
Underweight (BMI < 18.5)	0.33 (0.11, 0.99)	0.049	0.25 (0.08–0.83)	0.023
Normal weight (BMI 18.5–24.9)	Reference		Reference	–
Overweight (BMI 25–29.9)	4.02 (2.70, 6.00)	<.0001	3.91 (2.60–5.88)	<.0001
Obese (BMI ≥ 30)	4.98 (2.97, 8.35)	<.0001	4.78 (2.80–8.15)	<.0001
Maternal educational level		0.020		0.015
University and above	Reference		Reference-	
Junior college/Polytechnic	1.93 (1.28, 2.93)	0.002	1.96 (1.25–3.07)	0.004
ITE^c^	1.59 (0.88, 2.87)	0.128	1.45 (0.76–2.76)	0.262
Secondary and below	1.39 (0.88, 2.21)	0.162	1.05 (0.63–1.77)	0.853
Smoking before pregnancy, Y vs N	1.76 (1.14, 2.72)	0.011	1.67 (1.02–2.77)	0.043
Hypertensive disorders of pregnancy, Y vs N	2.74 (1.04, 7.20)	0.042	2.36 (0.79, 7.01)	0.117
4b) Logistic regression of predictors of total gestational weight gain *below* IOM recommendations				
Variable	Univariate OR ^d^		Multivariate Adjusted OR^e^	
	Odds ratio (95% CI)	P-value	Odds ratio (95% CI)	P-value
Maternal body mass index category (kg/m^2^)		<.0001		<.0001
Underweight (BMI < 18.5)	1.02 (0.59, 1.78)	0.945	1.06 (0.60–1.87)	0.841
Normal weight (BMI 18.5–24.9)	Reference-		Reference	
Overweight (BMI 25–29.9)	0.30 (0.19, 0.47)	<.0001	0.28 (0.18–0.44)	<.0001
Obese (BMI ≥ 30)	0.63 (0.37, 1.06)	0.079	0.60 (0.35–1.04)	0.067
Maternal age	1.03 (0.99, 1.06)	0.085	–	–
Marital status, Unmarried^f^ vs Married	0.55 (0.28,1.10)	0.090	–	–
Parity		0.020		0.002
0	Reference		Reference-	
1	1.63 (1.16, 2.29)	0.005	1.85 (1.29–2.64)	0.001
2 or more	1.28 (0.80, 2.06)	0.306	1.52 (0.98–2.69)	0.060
Smoking before pregnancy, Y vs N	0.69 (0.44, 1.08)	0.104	–	–
Smoking during pregnancy, Y vs N	0.31 (0.08, 1.24)	0.098	0.27 (0.06–1.21)	0.087
Exercise during pregnancy, Y vs N	0.58 (0.31, 1.07)	0.080	0.63 (0.33–1.19)	0.155

^a^Variables significant at p < 0.20 in univariate analysis; variables not listed include maternal age, race, parity, maternal employment at onset of pregnancy, monthly household income category, pre-existing hypertension, pre-existing diabetes, maternal thyroid disease, smoking during pregnancy, alcohol use before pregnancy, alcohol use during pregnancy, exercise before pregnancy, exercise during pregnancy and GDM

^b^Area under the curve = 0.732

^c^ITE stands for Institute of Technical Education, it is a public vocational education institution in Singapore that provides pre- employment training to secondary school leavers and continuing education and training to working adults

^d^Variables significant at *p* < 0.20 in univariate analysis; variables not listed include race, maternal educational level, maternal employment at onset of pregnancy, monthly household income category, pre-existing hypertension, pre-existing diabetes, maternal thyroid disease, alcohol use before pregnancy, alcohol use during pregnancy, exercise before pregnancy, hypertensive disorders of pregnancy and GDM

^e^Area under the curve = 0.643

^f^Unmarried include single, separated or divorced

Table 4 b shows the results of logistic regression on predictors of total GWG below IOM guidelines. In multivariate analysis, maternal BMI category was significantly associated with weight gain below the guidelines (*p* < .0001), with overweight women less likely to gain weight below IOM reference compared to normal weight women (adjusted OR: 0.28 [95% CI: 0.18–0.44]; *p* < .0001).

## Discussion

In our cohort, obese women as a group had a mean GWG that exceeded the upper limit of IOM 2009 GWG guidelines. Additionally, being overweight and obese significantly increased the risks of gaining gestational weight above IOM guidelines, compared to being normal weight.

In terms of percentages of women who gained weight within IOL recommendations, our cohort showed a higher adherence rate of 40.7% compared to a recent meta-analysis of more than one million pregnant women from diverse international cohorts which found that only 24.5% had pregnancy weight gain within IOM 2009 recommendations [1].

Within the different BMI subgroups, the observed weight gain patterns in our cohort are comparable to other Asian-centered population studies, with higher percentages of obese and overweight women gaining more weight than IOM guidelines and more underweight and normal weight women gaining weight below IOM recommendations. For example, Tanaka et al. [13] examined GWG in 1883 singleton low-risk Japanese women and found that 44% of the obese and 29.6% of the overweight women gained more weight than IOM guidelines, whereas 73.7% of underweight and 61.5% of normal weight women gained less weight than IOM guidelines. Similarly in another study, 10,973 singleton low-risk Taiwanese women that delivered after 37 weeks were analyzed; significantly higher proportions of obese (49.8%) and overweight (52.2%) pregnant mothers gained more weight than IOM recommendations, while higher proportions of underweight (44.4%) and normal weight (27.9%) women gained less weight than IOM recommendations [10]. Most available Asian studies, however, used IOM 2009 guidelines as a reference due to the lack of well-established GWG recommendations for Asian populations. The distributions of BMI categories, however, are different between Asian and the US populations, with a much lower prevalence of obesity in the Asian population. The prevalence of obesity among adult women in the US is estimated to be 41.1% in 2015–2016, with severe obesity (BMI ≥40 kg/m2) constitutes 9.7% of the population [17, 18]. The prevalence of obesity among American Asian women is 14.8% in the same time periods, much lower than the rest of ethnicity [17]. In contrast, the prevalence of obesity in the Singaporean women is estimated to be 7.9% [19], significantly lower than that for the overall adult American women and also lower than the rate in American Asian women. As weight gain recommendations may be affected by the baseline weight of a population, and the IOM recommendations use absolute values of weight based on data derived from American women who are in general higher in body weight than Asian women, it may be difficult to comment in these Asian studies whether gaining more weight than IOM guidelines indicate excessive weight gain and vice versa.

For predictors of GWG above IOM guidelines, overweight and obesity were found to be independent risk factors of gaining excessive gestational weight in our cohort. This is consistent with data from western countries [6, 7]. For example, in a study of 571 low-risk singleton pregnancies in the USA, overweight and obese women had significantly higher risks of total GWG above IOM guidelines, with adjusted OR of 3.44 and 4.55, respectively [7].

In the above cited studies, IOM 2009 GWG guidelines were used as a benchmark. Only one study made GWG recommendations based on data from Singaporean population. In 2014, Ee et al. studied the optimal GWG for Singaporeans based on the lowest aggregated risk for delivery mode and size for GA, with GWG value corresponding to vaginal delivery and baby size appropriate for GA defined as the optimal GWG [20]. Their analyses concluded that the optimal GWG for underweight Singaporean women may be higher than IOM guidelines with optimal GWG of 19.5 kg (range 12.9 to 23.9 kg); whereas the values for obese women should be less than IOM recommendations, with optimal GWG of 1.8 kg (range − 5.0 to 7.0 kg). Compared to their suggested values, the total GWG in our cohort was too low for the underweight and normal weight, and too high for obese women. The outcomes Ee’s study, however, were based only on delivery mode and size for GA and did not consider other maternal, perinatal and infant outcomes which were also taken into account by IOM 2009 GWG guidelines.

Several limitations of the current study merit attention. First, different BMI groups have different target weight gains. Hence pregnant women with a stricter target may empirically be more likely to fail to achieve it than a woman with a more lenient target, independently of BMI, and this may confound the relationship between BMI category and GWG. Second, BMI groups were classified according to WHO classification (instead of Asian BMI classifications). However, there are no available data that indicate a clear BMI cut-off point for Asians and WHO BMI cut-off values are widely used as international classifications [21]. Third, we compared the GWG of our cohort with IOM 2009 reference which is based on American population. The suitability of IOM 2009 GWG guidelines in Asian women have been investigated in many studies, but no consensus has been reached [9, 20, 22–24].

The effects of excessive weight gain in pregnancy have been extensively studied and linked to adverse maternal and neonatal outcomes [1, 25, 26]. As the implications of excessive GWG could be profound and with the rising prevalence of overweight and obesity in the Southeast Asian [27, 28], understanding the characteristics of GWG and making appropriate GWG advice for overweight and obese Singaporean women prior to and during pregnancy is important.

## Conclusions

In our cohort, obese women as a group had a mean total GWG that exceeded the upper limit of IOM recommendations. Overweight and obesity are independent significant risk factors of gaining excessive gestational weight. Our study adds to the understanding of the characteristics of pregnancy weight gain in the Singaporean population, and suggests that appropriate weight management for overweight and obese women prior to and during pregnancy is important. The implications of our results on pregnancy outcomes could be investigated in future studies. In addition, high quality studies with large sample sizes are needed to develop GWG recommendations in the multi-ethnic Singaporean populations.

## Additional file

Additional file 1: **Table S1.** Baseline characteristics of each BMI groups. (DOCX 19 kb)

## Data Availability

All data generated or analysed during this study are included in this published article [and its supplementary information files].

## Abbreviations

BMI: Body Mass Index
GA: Gestation Age
GWG: Gestational Weight Gain
IOM: Institute of Medicine
NORA: Neonatal and Obstetric Risk Assessment
WHO: World Health Organization

## References

[1] Goldstein RF, Abell SK, Ranasinha S, Misso M, Boyle JA, Black MH (2017). Association of gestational weight gain with maternal and infant outcomes: a systematic review and meta-analysis. JAMA.

[2] Siega-Riz AM, Viswanathan M, Moos M-K, Deierlein A, Mumford S, Knaack J (2009). A systematic review of outcomes of maternal weight gain according to the Institute of Medicine recommendations: birthweight, fetal growth, and postpartum weight retention. Am J Obstet Gynecol.

[3] Obstetricians AC of, Gynecologists, others. ACOG (2013). Committee opinion no. 548: weight gain during pregnancy. Obstet Gynecol.

[4] Rasmussen KM, Yaktine AL, Institute of Medicine (US) and National Research Council (US) Committee to Reexamine IOM Pregnancy Weight Guidelines (2009). Weight Gain During Pregnancy: Reexamining the Guidelines [Internet].

[5] Brawarsky P, Stotland NE, Jackson RA, Fuentes-Afflick E, Escobar GJ, Rubashkin N (2005). Pre-pregnancy and pregnancy-related factors and the risk of excessive or inadequate gestational weight gain. Int J Gynaecol Obstet Off Organ Int Fed Gynaecol Obstet.

[6] Godoy AC, Do NSL, Surita FG (2015). A systematic review and meta-analysis of gestational weight gain recommendations and related outcomes in Brazil. Clinics (Sao Paulo).

[7] Rosal MC, Wang ML, Moore Simas TA, Bodenlos JS, Crawford SL, Leung K (2016). Predictors of gestational weight gain among white and Latina women and associations with birth weight. J Pregnancy.

[8] Stotland NE, Cheng YW, Hopkins LM, Caughey AB (2006). Gestational weight gain and adverse neonatal outcome among term infants. Obstet Gynecol.

[9] Enomoto K, Aoki S, Toma R, Fujiwara K, Sakamaki K, Hirahara F (2016). Pregnancy outcomes based on pre-pregnancy body mass index in Japanese women. PLoS One.

[10] Hung T-H, Hsieh T-T (2016). Pregestational body mass index, gestational weight gain, and risks for adverse pregnancy outcomes among Taiwanese women: a retrospective cohort study. Taiwan J Obstet Gynecol.

[11] Suzuki S (2017). Gestational weight gain in Japanese women with favorable perinatal outcomes. J Clin Med Res.

[12] Suzuki S (2016). Optimal weight gain during pregnancy in Japanese women. J Clin Med Res.

[13] Tanaka T, Ashihara K, Nakamura M, Kanda T, Fujita D, Yamashita Y (2014). Associations between the pre-pregnancy body mass index and gestational weight gain with pregnancy outcomes in Japanese women. J Obstet Gynaecol Res.

[14] Xiong C, Zhou A, Cao Z, Zhang Y, Qiu L, Yao C (2016). Association of pre-pregnancy body mass index, gestational weight gain with cesarean section in term deliveries of China. Sci Rep.

[15] Lim WY, Saw SM, Tan KH, Yeo GS, Kwek KY (2012). A cohort evaluation on arterial stiffness and hypertensive disorders in pregnancy. BMC Pregnancy Childbirth.

[16] Cedergren MI (2007). Optimal gestational weight gain for body mass index categories. Obstet Gynecol.

[17] The state of obesity 2018: Better policies for a healthier America [internet]. Self-sufficiency research clearinghouse. [cited 2019 Feb 6]. Available from: https://www.opressrc.org/content/state-obesity-2018-better-policies-healthier-america

[18] Hales CM, Fryar CD, Carroll MD, Freedman DS, Ogden CL (2018). Trends in obesity and severe obesity prevalence in US youth and adults by sex and age, 2007-2008 to 2015-2016. JAMA..

[19] Lee YS, Biddle S, Chan MF, Cheng A, Cheong M, Chong YS (2016). Health promotion board–Ministry of Health clinical practice guidelines: obesity. Singap Med J.

[20] Ee TX, Allen JC, Malhotra R, Koh H, Østbye T, Tan TC (2014). Determining optimal gestational weight gain in a multiethnic Asian population. J Obstet Gynaecol Res.

[21] Expert Consultation WHO (2004). Appropriate body-mass index for Asian populations and its implications for policy and intervention strategies. Lancet Lond Engl.

[22] Du M-K, Ge L-Y, Zhou M-L, Ying J, Qu F, Dong M-Y (2017). Effects of pre-pregnancy body mass index and gestational weight gain on neonatal birth weight. J Zhejiang Univ Sci B.

[23] Yang S, Peng A, Wei S, Wu J, Zhao J, Zhang Y (2015). Pre-pregnancy body mass index, gestational weight gain, and birth weight: a cohort study in China. PLoS One.

[24] Liu Y, Dai W, Dai X, Li Z (2012). Prepregnancy body mass index and gestational weight gain with the outcome of pregnancy: a 13-year study of 292,568 cases in China. Arch Gynecol Obstet.

[25] Rong K, Yu K, Han X, Szeto IMY, Qin X, Wang J (2015). Pre-pregnancy BMI, gestational weight gain and postpartum weight retention: a meta-analysis of observational studies. Public Health Nutr.

[26] Fuemmeler BF, Wang L, Iversen ES, Maguire R, Murphy SK, Hoyo C (2016). Association between Prepregnancy body mass index and gestational weight gain with size, tempo, and velocity of infant growth: analysis of the newborn epigenetic study cohort. Child Obes.

[27] Angkurawaranon C, Jiraporncharoen W, Chenthanakij B, Doyle P, Nitsch D (2014). Urban environments and obesity in Southeast Asia: a systematic review, meta-analysis and meta-regression. PLoS One.

[28] Jan Mohamed HJB, Yap RWK, Loy SL, Norris SA, Biesma R, Aagaard-Hansen J (2015). Prevalence and determinants of overweight, obesity, and type 2 diabetes mellitus in adults in Malaysia. Asia Pac J Public Health.

