# Supplementary material for: Are women in Singapore gaining weight appropriately during pregnancy: a prospective cohort study
Source: BMC Pregnancy Childbirth. 2019 Aug 13;19:290. doi: 10.1186/s12884-019-2443-z (PMC6693141; doi:10.1186/s12884-019-2443-z)
Supplement: Supplementary file 1 — Table S1. Baseline characteristics of each BMI groups. (DOCX 19 kb) [file 12884_2019_2443_MOESM1_ESM.docx]

**Table S1 Baseline characteristics of each BMI groups**

| **Characteristics** | **Under-weight**  **(N=59)** | **Normal weight (N=414)** | **Over-weight**  **(N=173)** | **Obese**  **(N=78)** | **P value** |
| --- | --- | --- | --- | --- | --- |
| *Demographic factors* |  |  |  |  |  |
| Maternal age (year), mean (SD) | 28.8 (5.4) | 30.3 (4.6) | 31.3 (5.2) | 31.6 (5.2) | <.0001 |
| Maternal age range (year), n (%) |  |  |  |  |  |
| < 25 | 14 (18.2) | 44 (57.1) | 13 (16.9) | 6 (7.8) | <.0001 |
| 25 - 29 | 20 (8.5) | 142 (60.4) | 54 (23.0) | 19 (8.1) |  |
| 30 - 34 | 19 (7.7) | 152 (61.3) | 48 (19.4) | 29 (11.7) |  |
| 35 - 40 | 4 (2.9) | 66 (48.2) | 50 (36.5) | 17 (12.4) |  |
| > 40 | 2 (7.4) | 10 (37) | 8 (29.6) | 7 (25.9) |  |
| Maternal body mass index (kg/m^2^), mean (SD) | 17.5 (0.9) | 21.7 (1.7) | 27.2 (1.5) | 33.2 (3.1) | NA |
| Race, n (%) |  |  |  |  |  |
| Chinese | 37 (9.8) | 257 (68.0) | 63 (16.7) | 21 (5.6) | <.0001 |
| Malay | 13 (10.8) | 73 (60.8) | 26 (21.6) | 8 (6.7) |  |
| Indian | 4 (2.8) | 33 (23.4) | 68 (48.2) | 36 (25.5) |  |
| Others | 5 (5.9) | 51 (60.0) | 16 (18.8) | 13 (15.3) |  |
| Parity, n (%) |  |  |  |  |  |
| 0 | 43 (10.6) | 245 (60.6) | 80 (19.8) | 36 (8.9) | <.0001 |
| 1 | 16 (7.1) | 126 (56.0) | 63 (28.0) | 20 (8.9) |  |
| 2 or more | 0 (0) | 43 (45.3) | 30 (31.6) | 22 (23.2) |  |
| Marital status, n (%) |  |  |  |  |  |
| Married | 52 (7.7) | 385 (57.1) | 163 (24.2) | 74 (11.0) | 0.06 |
| Single | 7 (15.2) | 28 (60.9) | 9 (19.6) | 2 (4.3) |  |
| Separated/Divorced | 0 (0) | 1 (25) | 1 (25) | 2 (50) |  |
| Maternal employment at onset of pregnancy, n (%) |  |  |  |  |  |
| Employed | 46 (7.9) | 338 (58.2) | 138 (23.8) | 59 (10.2) | 0.62 |
| Unemployed | 13 (9.1) | 76 (53.1) | 35 (24.5) | 19 (13.3) |  |
| Maternal educational level |  |  |  |  |  |
| Secondary and below | 15 (9.1) | 82 (50) | 39 (23.8) | 28 (17.1) | 0.158 |
| ITE ^a^ | 7 (9.6) | 41 (56.2) | 15 (20.5) | 10 (13.7) |  |
| Junior college/Polytechnic | 17 (8.1) | 122 (57.8) | 53 (25.1) | 19 (9.0) |  |
| University and above | 20 (7.3) | 168 (61.3) | 65 (23.7) | 21 (7.7) |  |

**Table S1 Baseline characteristics of each BMI groups (continued)**

| **Characteristics** | **Under-weight**  **(N=59)** | **Normal weight (N=414)** | **Over-weight**  **(N=173)** | **Obese**  **(N=78)** | **P value** |
| --- | --- | --- | --- | --- | --- |
| Total monthly household income ^b^, n (%) |  |  |  |  |  |
| ≤1300 | 3 (12.5) | 14 (58.3) | 3 (12.5) | 4 (16.7) | 0.004 |
| 1301-3500 | 22 (9.7) | 105 (46.3) | 65 (28.6) | 35 (15.4) |  |
| 3501-5500 | 16 (7.3) | 139 (63.8) | 39 (17.9) | 24 (11.0) |  |
| 5500-8500 | 10 (6.1) | 98 (60.1) | 46 (28.2) | 9 (5.5) |  |
| ≥8501 | 8 (8.9) | 57 (63.3) | 19 (21.1) | 6 (6.7) |  |
| *Pre-pregnancy medical conditions* |  |  |  |  |  |
| Pre-existing hypertension, n (%) | 0 (0) | 2 (22.2) | 3 (33.3) | 4 (44.4) | 0.006 |
| Pre-existing diabetes mellitus, n (%) | 0 (0) | 5 (41.7) | 6 (50.0) | 1 (8.3) | 0.168 |
| Thyroid disease, n (%) | 0 (0) | 12 (66.7) | 5 (27.8) | 1 (5.6) | 0.498 |
| *Behavioral factors* |  |  |  |  |  |
| Smoking, n (%) |  |  |  |  |  |
| Before current pregnancy | 14 (12.8) | 48 (44.0) | 29 (26.6) | 18 (16.5) | 0.009 |
| During current pregnancy | 1 (5.9) | 8 (47.1) | 4 (23.5) | 4 (23.5) | 0.384 |
| Alcohol use, n (%) |  |  |  |  |  |
| Before current pregnancy | 26 (11.9) | 145 (66.2) | 35 (16.0) | 13 (5.9) | <.0001 |
| During current pregnancy | 1 (11.1) | 8 (88.9) | 0 (0) | 0 (0) | 0.182 |
| Exercise, n (%) |  |  |  |  |  |
| Before current pregnancy | 17 (5.0) | 198 (58.1) | 93 (27.3) | 33 (9.7) | 0.008 |
| During current pregnancy | 1 (1.6) | 30 (49.2) | 21 (34.4) | 9 (14.8) | 0.041 |

^a^ ITE stands for Institute of Technical Education, it is a public vocational education institution in Singapore that provides pre-employment training to secondary school leavers and continuing education and training to working adults.

^b^ Total monthly household income is measured in Singapore dollars, is defined as the combined monthly income for married couple, or patient’s monthly income if she is single, separated or divorced.
